# 小分子靶标的非固定化核酸适配体筛选技术研究进展

**DOI:** 10.3724/SP.J.1123.2024.04012

**Published:** 2025-04-08

**Authors:** Yangyang HU, Ge YANG, Feng QU

**Affiliations:** 1.北京理工大学生命学院,北京 100081; 1. School of Life Science, Beijing Institute of Technology, Beijing 100081, China; 2.中国医学科学院北京协和医学院医药生物技术研究所,北京 100050; 2. Institute of Medicinal Biotechnology, Chinese Academy of Medical Sciences and Peking Union Medical College, Beijing 100050, China

**Keywords:** 指数富集配体系统进化技术, 小分子靶标, 非固定化核酸适配体筛选技术, 综述, systematic evolution of ligands by exponential enrichment (SELEX) techniques, small molecule targets, non-immobilized aptamer screening techniques, review

## Abstract

核酸适配体是通过指数富集配体系统进化(SELEX)技术获得的一段核糖核酸(RNA)或单链脱氧核糖核酸(ssDNA),可用于靶标的特异性识别。核酸适配体具有易于化学合成和修饰、高热稳定性、低毒性与低免疫原性等优势。小分子靶标的核酸适配体在新药研发、肿瘤治疗、疾病诊断、超快速灵敏检测、环境污染监测和毒品检测等领域具有广泛的应用前景。然而,小分子因结构简单、分子质量小且可供核酸结合的基团有限,导致靶向小分子的适配体结合不稳定,为适配体的筛选与传感器的开发带来了巨大挑战。高效的筛选技术是获取高性能适配体的关键,目前适用于小分子靶标的核酸适配体筛选技术主要分为3类:基于靶标固定的筛选技术、基于核酸库固定的筛选技术以及靶标非固定化的筛选技术。与其他两种筛选技术相比,靶标非固定化的筛选技术所需的筛选轮数更少且获得的适配体亲和力更高(通常在nmol/L水平)。本文总结了小分子靶标的非固定化核酸适配体筛选技术(包括氧化石墨烯(GO)-SELEX、毛细管电泳(CE)-SELEX和纳米金辅助(GNP)-SELEX )的原理、优缺点及应用进展。此外,本文总结了在适配体特异性评价中对照靶标的选择策略。

## Abstract (en)

Aptamers obtained through systematic evolution of ligands by exponential enrichment (SELEX) techniques are single stranded deoxyribonucleic acid (ssDNA) or RNA molecules capable of specifically recognizing target molecules. Such aptamers are easily chemically synthesized and modified, highly thermally stable, and are low toxicity and low immunogenicity. Aptamers that target small molecules have broad applications prospects for the development of new drugs, treating tumors, diagnosing diseases, monitoring environmental pollution, detecting drugs, and in ultrafast and sensitive detection applications. However, the simple structures and low molecular masses of small molecules, along with the limited number of binding groups available for interacting with nucleic acids lead to unstable aptamer-small molecule binding, which poses significant challenges for aptamer screening and sensor development. Efficient screening techniques are crucial for identifying aptamers with excellent performance characteristics. At present, the aptamer screening techniques suitable for small-molecule targets are mainly divided into three categories: target-immobilized-based screening technique, nucleic acid library-immobilized-based screening technique, and target-non-immobilized screening technique. Among them, target-non-immobilized screening technique require fewer screening rounds and result in aptamers with superior (typically nmol/L level) affinities. This paper summarized non-immobilized aptamer screening techniques for small-molecule targets, including principle, advantages, disadvantages and application progress associated with graphene oxide (GO)-SELEX, capillary electrophoresis (CE)-SELEX, and gold nanoparticle-assisted (GNP)-SELEX techniques. In addition, strategies for selecting control targets in aptamer-specific evaluation were summarized.

核酸适配体(aptamer)是通过指数富集配体系统进化(systematic evolution of ligands by exponential enrichment, SELEX)技术^[1]^获得的一段核糖核酸(ribonucleic acid, RNA)或单链脱氧核糖核酸(single stranded deoxyribonucleic acid, ssDNA),具有特异性的分子识别功能。与抗体相比,核酸适配体具有易于化学合成和修饰、高热稳定性、低毒性与低免疫原性等优点;其中,小分子靶标的核酸适配体在新药研发、肿瘤治疗、疾病诊断、超快速灵敏检测、环境污染监测和毒品检测等领域具有广泛的应用前景^[2⇓⇓⇓⇓-7]^。目前,已有100余种靶向小分子的适配体被筛选出来^[8]^,将这些小分子的适配体与比色法、表面等离子体共振(surface plasmon resonance, SPR)、电化学、光学、化学发光免疫和原位滚环扩增(rolling circle amplification, RCA)等技术结合,设计出了各种类型的适配体传感器。然而,小分子的结构简单、分子质量小且可供核酸结合的基团有限,仅有少数适配体传感器能够满足实际应用所需的灵敏度和特异性要求^[7]^,这为适配体的筛选和传感器的开发带来了巨大挑战。

高效的筛选技术是获得高性能适配体的关键^[9]^。自适配体概念提出30多年来,已有10多种改良型SELEX技术相继被报道,其中适用于小分子靶标的适配体筛选技术主要分为3类:基于靶标固定的筛选技术、基于核酸库固定的筛选技术以及靶标非固定化的筛选技术。基于靶标固定的筛选技术通常需要将靶标偶联到固相介质表面,以此来分离候选适配体与核酸库中未与靶标结合的序列。然而,小分子的结构简单且官能团较少,其固定化过程较为困难。与蛋白质不同,小分子无法通过1-乙基-3-(3-二甲基氨基丙基)-碳二亚胺(1-ethyl-3-(3-dimethylaminopropyl)-carbodiimide, EDC)和*N*-羟基琥珀酰亚胺(*N*-hydroxysuccinimide, NHS)等多种标准化学方法来实现固定^[10⇓⇓-13]^。此外,即便小分子被固定,固相介质也会对小分子的官能团产生掩蔽效应,进而改变其理化性质,这严重限制了小分子与适配体的有效结合^[7]^。有研究^[14]^表明,基于靶标固定的筛选技术(如偶联小分子)获得的适配体对自由靶标的亲和力大大降低或完全丧失。与基于靶标固定的筛选技术相比,近年来基于核酸库固定的筛选技术得到了更为广泛的应用,其中捕获(Capture)-SELEX技术是这一领域的典型代表。贾海静等^[9]^基于Capture-SELEX技术,考察了36种靶标的实验条件,并分析了所获适配体的亲和力与特异性,探讨了实验条件与适配体性能之间的关系。同时,贾海静等^[9]^还以氧化石墨烯(GO)-SELEX技术作为参照,对比了近三年采用靶标非固定化的筛选技术所获得的13种小分子靶标适配体的实验条件及适配体性能。结果表明,与基于核酸库固定的筛选技术相比,靶标非固定化的筛选技术所需的筛选轮数更少,且获得的适配体亲和力更高(通常在nmol/L水平)。因此,使用靶标非固定化的筛选技术,有望解决因靶标或核酸库固定而导致的适配体亲和力下降的问题。

近年来,针对小分子靶标的非固定化核酸适配体筛选技术发展迅速,主要包括以下3种:GO-SELEX技术、毛细管电泳(CE)-SELEX技术和纳米金辅助(GNP)-SELEX技术。在Web of Science数据库中,通过输入“GO”“CE”“GNP”“非固定化筛选技术”这4种关键词中的任意一种,并结合“小分子”与“适配体”两个关键词进行检索,共可检索到45篇与小分子靶标的非固定化核酸适配体筛选技术相关的研究论文;其中,2005-2011年发表了4篇,2012-2017年发表了18篇,2018-2023年发表了23篇。本文主要对GO-SELEX技术、CE-SELEX技术和GNP-SELEX技术的原理、优缺点及研究进展进行了总结,探讨了在适配体特异性评价中对照靶标的选择策略。

## 1 GO-SELEX技术

### 1.1 GO-SELEX技术的介绍

GO-SELEX是一种以GO为靶标-适配体复合物分离介质的筛选技术。GO是一种新型的二维碳纳米材料,它能够通过核酸碱基与GO六边形单元之间的*π*-*π*堆积作用来吸附ssDNA或RNA,而双链DNA因其碱基被包裹在双螺旋结构内部,不会被GO吸附。在筛选过程中,GO能够有效吸附游离核酸库中的ssDNA,而较难吸附与靶标结合的核酸序列,因此GO可用于去除那些未与靶标结合的游离ssDNA。同时,GO在水溶液中具有良好的分散性和亲水性,能够有效降低表面自由能,是理想的非固定化筛选分离介质。GO-SELEX技术无需固定靶标,因此避免了靶标构象的变化,是一种简单、快速的适配体筛选方法。

 2012年,Park等^[15]^首次提出了GO-SELEX技术,并成功筛选出了烟酰胺磷酸核糖转移酶(nicotinamide phosphoribosyl transferase, NAMPT)的适配体。目前,GO-SELEX技术已成功应用于多种小分子靶标(包括农药、毒素、抗生素、药物、生物代谢物、有机污染物)的DNA适配体筛选^[16⇓⇓⇓⇓⇓⇓⇓⇓⇓⇓⇓⇓⇓⇓⇓⇓⇓⇓⇓-36]^,所获得的适配体序列、筛选轮数及平衡解离常数(*K*_D_)等见表1。以*K*_D_来衡量小分子靶标与适配体之间的亲和力,*K*_D_值越小,表示靶标与适配体之间的亲和力越高;反之,*K*_D_值越大,亲和力越低。如表1所示,经过4~16轮筛选后,所获得的*K*_D_为0.28~260.10 nmol/L。

**表1:** GO-SELEX技术在小分子靶标适配体筛选中的应用

Type	Targets	Aptamer sequences (5'-3')	Screening round	Affinity determina- tion methods	*K*_D_/ (nmol/L)
Pesticides	tebuconazole, inaben- fide, mefenacet^[16]^	CGTACGGAATTCGCTAGCAGCGTCCACGAGTGTGG- TGTGGATCCGAGCTCCACGTG, etc.	5	isothermal titration calorimetry (ITC)	10.00-100.00
	glyphosate (GlyP)^[17]^	GGACAGCTGGCCGCGTAGCGAGACACGTACAAGG- TACTATACGGCTGGCATATGTATCTG	8	fluorescence assay	30.73±1.25^*^
	thiamethoxam (TMX)^[18]^	GTCGACGGATCCACCGACCATGCAAAGATGCACAA- AAACGACAGCAGCTGCAGGTCGAC	9	gold nanoparticle colorimetric assay	118.34±13.85^*^
Toxins	T2 toxin^[19]^	GTATATCAAGCATCGCGTGTTTACACATGCGAGAG- GTGAA	10	fluorescence assay	20.80±3.10^*^
	gonyautoxin 1/4 (GTX1/4)^[20]^	AACCTTTGGTCGGGCAAGGTAGGTT	8	biolayer interfer- ometry (BLI)	21.90
	domoic acid (DA)^[21]^	AAAAATAATTTAAATTTTCTACCCAATGCTTTTCGC- ATAA	16	fluorescence assay	62.07±19.97^*^
	tetrodotoxin (TTX)^[21]^	TCAAATTTTCGTCTACTCAATCTTTCTGTCTTATC	16	fluorescence assay	44.12±15.38^*^
	saxitoxin (STX)^[21]^	CTTTTTACAAAATTCTCTTTTTACCTATATTATGAA- CAGA	16	fluorescence assay	61.44±23.18^*^
	gliotoxin^[22]^	CATGTGTCCACATGGAGGTGACCT	8	BLI, ITC	196.00
	okadaic acid^[23]^	ATTTGACCATGTCGAGGGAGACGCGCAGTCGCTAC- CACCT	8	fluorescence assay	5.63±3.55^*^
Antibiotics	penicillin G (Pen G)^[24]^	CACCAGTCAGACAGCACGGTGACTGGAGTGACGTC- GGTACCTGAGATCGAGTGACGTCGGTACCTG	9	electrochemical sensing	105.15±1.94^*^
	patulin^[25]^	GGCCCGCCAACCCGCATCATCTACACTGATATTTTA- CCTT	15	fluorescence assay	21.83±5.02^*^
	ofloxacin^[26]^	TGGCGCTTAGGTGTAATAACCTGAGGACGGCTTGG, TGGTTAAACCACGGTGAACCACTGCGCAGTAGGTC	7	balanced filtration	130.10, 159.10
Drugs	ractopamine (RAC)^[27]^	AGCAGCACAGAGGTCAGATGGTCTCTACTAAAAGT- TTTGATCATACCGTTCACTAATTGACCTATGCGTGC- TACCGTGAA	16	GO adsorption	54.22±8.02^*^
	synthetic cannabinoids (SCs)^[28]^	C6-NH2-AGGAATTCAGATCTCCCTGCAGTGGTGTTC- AATGTTTTTGTGCTGTTCTGTACTGGCGCCTCGAGG- AGCTCAGGATCCCG-SH	5	electrochemical sensing	-
	sulfamethazine (SMZ)^[29]^	CGTTAGACG	7	fluorescence assay	24.60
	ephedrine^[30]^	TCCGTCGGCGGCGGCCCCTTCCTACAGCTTTCCCG- GTCGC	10	ITC	2.86±0.24^*^
	tramadol (TH)^[31]^	CTTAAACCTGGTCGGATAGTCTTCGAGACTCGCGG- TCGCATTT	10	fluorescence assay	178.40
	L-carnitine^[32]^	ACCTTGCGTGCTCACGGCAGCCTCTCGGACAGCCC- TGTGT	13	resonance rayleigh scattering (RRS)	-
Biological metabolites	*β*-carotene^[33]^	CAGCTCAGAAGCTTGATCCTCCCACAATTATCACGT- AGTGTGCGGGTCACGCAATCTGACGACTCGAAGTC- GTGCATCTG	6	fluorescence assay	5.04±1.99^*^
	25-hydroxyvitamin D3^[34]^	AGCAGCACAGAGGTCATGGGGGGTGTGACTTTGGT- GTGCCTATGCGTGCTACGGAA	4	ITC	11.00
	sarcosine^[35]^	TAGGGAAGAGAAGGACATATGATGTGCCGCGCTT- CCCTTGCCGCTCAAAACAGACCACCCACTTTGACT- AGTACATGACCACTTGA	8	fluorescence assay	0.33±0.05^*^
Organic pollutant	nonylphenol (NP)^[36]^	ATGCGGATCCCGCGCGGCCGGCCAGTGCGCGAAG- CTTGCGC	5	gold nanoparticle colorimetric assay	194.20±65.90^*^

*K*_D_: equilibrium dissociation constants; *: *n*=3; -: not given.

### 1.2 GO-SELEX技术的优势与应用

GO-SELEX技术可用于混合靶标的正向与反向筛选,从而成功筛选出多个靶标的适配体。多重(multiple) GO-SELEX技术能够利用GO来筛选出一组针对混合小分子靶标的适配体,是一种简单、快速且高通量的适配体筛选方法。如Nguyen等^[16]^利用multiple GO-SELEX技术,通过正向与反向筛选,成功实现了多个靶标适配体的同时筛选。在反向筛选过程中,首先将6种不同的反筛靶标与核酸库混合,然后加入GO分散液,经过1 h的孵育后,通过离心来去除与反筛靶标结合的ssDNA。在正向筛选过程中,将结合了反筛靶标-ssDNA复合物的GO与戊唑醇、抗倒胺、苯噻草胺3种混合靶标进行孵育,使ssDNA的结构发生改变,从而导致其从GO表面脱附,并与相应的靶标结合,形成靶标-ssDNA复合物。经过正向筛选后,靶标-ssDNA复合物会被释放回溶液中,随后再利用聚合酶链式反应(polymerase chain reaction, PCR)、聚丙烯酰胺凝胶电泳(polyacrylamide gel electrophoresis, PAGE)及测序技术,筛选出10种不同的靶标适配体,所获得的*K*_D_为10~100 nmol/L。该研究不仅为3种靶标(戊唑醇、抗倒胺、苯噻草胺)分别筛选出了3个特异性适配体,而且还发现了能够同时靶向2个或3个不同靶标的适配体。对多个靶标的适配体进行同时筛选,不仅为适配体在分子诊断和生物技术领域的发展带来了新机遇,还为基于适配体的生物传感器应用开辟了广阔的前景。

采用GO-SELEX技术有助于获得高亲和力的适配体。Gu等^[23]^分别使用磁珠(MB)-SELEX技术和GO-SELEX技术对冈田酸的适配体进行筛选,结果表明,使用GO-SELEX技术所获得的*K*_D_((5.63±3.55) nmol/L)约为MB-SELEX技术(*K*_D_为(134.8±1.12) nmol/L)的1/24,说明亲和力提高了约24倍。Gao等^[20]^分别使用GO-SELEX技术和MB-SELEX技术对膝沟藻毒素1/4 (GTX1/4)的适配体进行筛选,并在实验条件尽可能一致的前提下,对比了两种技术的筛选效果。结果表明,通过GO-SELEX技术筛选获得的6个适配体均表现出了极高的亲和力,*K*_D_为62~653 nmol/L,而通过MB-SELEX技术筛选得到的适配体中,仅有3个序列能够与靶标结合,且*K*_D_处于μmol/L水平。与MB-SELEX技术相比,GO-SELEX在ssDNA回收率、序列同源性和适配体亲和力等方面都表现出了更大的优势;并且,GO-SELEX技术的筛选效率更高,能够快速吸附未结合的ssDNA,极大地简化了分离步骤,使得操作更为便捷。

基于GO-SELEX技术筛选出的适配体已应用于多个领域,如药物靶向传输、肿瘤细胞检测、病原体或生化指标检测及农药检测等。在药物靶向传输方面,Mo等^[37]^开发了一种以DNA-GO纳米聚集体(DNA-GA)为载体的5'-三磷酸腺苷(adenosine-5'-triphosphate, ATP)响应型抗癌药物递送策略,并成功用于控制小分子抗癌药物多柔比星(doxorubicin, DOX)的释放。DNA-GA由GO、两个ssDNA (DNA1和DNA2)和ATP的适配体组成。利用GO与DOX之间的超分子*π*-*π*堆叠作用,DNA-GA的分层结构可以高效率地负载DOX,形成DOX/DNA-GA复合物。在此过程中,DNA-GA的平均尺寸增大,比表面积减小,这有助于抑制GO中DOX的释放。当ATP存在时,ATP会与其适配体结合,导致DOX从DOX/DNA-GA复合物中解离。因此,与缺乏ATP的细胞外液相比,高ATP水平的环境(如细胞质)更能促进DOX从DOX/DNA-GA复合物中释放。该研究结果表明,通过DNA-GA与体内特定内环境之间的作用,可以精准控制药物的释放,从而实现药物在特定细胞内的按需靶向递送。在肿瘤细胞检测方面,GO与适配体的缀合物能够从血液中靶向捕获肿瘤细胞,随后采用特殊成像方法对捕获的肿瘤细胞进行分析,可用于肿瘤疾病的早期诊断^[19,38]^。基于此,Pramanik等^[39]^报道了一种双光子发光平台,该平台由S6 RNA适配体与GO共轭构成,可用于乳腺癌细胞(SK-BR-3)的靶向生物成像。当GO与S6 RNA适配体结合后,展现出极高的双光子吸收特性。当S6 RNA适配体与SK-BR-3靶向结合后,通过近红外光的照射,能够激发出强烈的双光子荧光信号,由此可以区分靶向的SK-BR-3与其他非靶向细胞。在病原体或生化指标检测方面,将GO-适配体与其他纳米材料联合用于生物传感器的制备^[40,41]^,能够显著提升传感器在生物检测应用中的灵敏度。He等^[42]^报道了一种基于GO-适配体的高灵敏度电化学检测平台,用于凝血酶的检测。在该平台中,凝血酶的适配体被固定在电极的一侧,电化学氧化还原探针被固定在电极的另一侧。当凝血酶存在时,固定在电极上的适配体会与其结合,形成电子屏障,阻碍电子传递,从而导致固定在电极另一侧的探针所产生的电化学信号减弱。此外,GO的不导电性进一步加剧了探针电化学信号的降低。因此,通过监测凝血酶识别前后电化学检测平台信号的变化,可以实现凝血酶的高灵敏度检测。结果表明,该方法对凝血酶具有较高的灵敏度(检出限(LOD)为1 pmol/L)和较宽的线性范围(0.005~50 nmol/L)。在农药检测方面,Kong等^[18]^采用GO-SELEX技术,经过正向与反向筛选,共筛选出了5个具有代表性的噻虫嗪(thiamethoxam, TMX)适配体。选择亲和力最高的适配体seq.20 (*K*_D_为(210.47±79.37) nmol/L)进行截短处理,截短后的适配体(seq.20-1和seq.20-2)均表现出了更高的亲和力(*K*_D_分别为(118.34±13.85) nmol/L和(123.35±29.80) nmol/L)。研究将截短后的适配体seq.20-2和原始适配体seq.20用作识别元件,构建了基于金纳米颗粒的比色适配体传感器,用于蔬菜样品中TMX的检测。结果发现,适配体seq.20-2对TMX的检测灵敏度(LOD为(1.67±0.12) nmol/L,信噪比(*S/N*)=3)优于适配体seq.20 (LOD为(3.33±0.23) nmol/L, *S/N*=3),这一结果证实了截短后的适配体作为识别元件在检测TMX中的可行性。

### 1.3 GO-SELEX技术存在的问题与挑战

使用GO-SELEX技术筛选小分子靶标的适配体时,主要存在下述问题与挑战:(1)在实验过程中,pH和温度的变化或某些离子的存在都可能削弱ssDNA与GO表面之间的结合力,使得ssDNA自发地从GO表面解吸附,进而降低筛选效率。此外,如果小分子靶标被GO吸附,那么利用GO-SELEX技术将无法有效筛选出针对该小分子靶标的适配体。(2)GO-SELEX技术依赖于GO的特定理化性质,因此制备具有单一理化性质的GO是确保GO-SELEX技术稳定性和应用准确性的关键。(3)在适配体筛选过程中,GO和小分子靶标均与适配体之间存在亲和力,如何增加这两种亲和力之间的差异,也是一个需要解决的问题。(4)有研究^[43,44]^表明,GO具有一定的细胞毒性,这可能会限制其在临床上的进一步应用。

## 2 CE-SELEX技术

### 2.1 CE-SELEX技术的介绍

CE-SELEX技术能够在自由溶液中促进靶标与核酸库的相互作用,并有效地将迁移率存在明显差异的靶标-核酸复合物与未结合的核酸分离开;此外,该技术还能够在毛细管的出口端定时、精确地收集靶标-核酸复合物。一般来说,采用CE-SELEX技术仅需1~4轮筛选,即可获得蛋白质等生物分子的适配体。在适配体筛选过程中,CE-SELEX技术能够保持靶标与核酸库的天然结构,并可以模拟生物体的内环境,是最理想的适配体筛选方法^[45]^。

目前,CE主要有7种基础分离模式^[46⇓⇓⇓⇓⇓⇓-53]^,其中被应用于CE-SELEX技术的分离模式有3种,分别为毛细管区带电泳(capillary zone electrophoresis, CZE)^[46,47]^、亲和毛细管电泳(affinity capillary electrophoresis, ACE)^[50]^和毛细管等速电泳(capillary isotachophoresis, CITP)^[53]^。在此基础上,多种CE-SELEX筛选模式得以发展,以下将重点介绍7种常见的筛选模式。(1)平衡混合物的非平衡毛细管电泳(NECEEM)-SELEX^[54⇓-56]^:首先将靶标与核酸库共同孵育,待形成“结合-解离”平衡混合物后,再将其注入毛细管柱中进行复合物的分离与收集。(2)低pH(LpH)-CE-SELEX^[57]^: CZE背景缓冲液的pH<3,使得靶标-核酸库混合物中的游离核酸与复合物的迁移方向相反(即游离核酸向进样端迁移),从而实现复合物的高效分离。(3)非(Non)-SELEX^[58⇓-60]^:在CZE-SELEX筛选过程中,轮次间不进行次级库的扩增与纯化步骤,而是将上一轮筛选后收集的复合物组分直接与靶标混合,并进行下一轮的CZE筛选。(4)同步竞争毛细管电泳(scCE)-SELEX^[60]^:将核酸库与两种靶标进行共孵育,两种靶标会各自特异性地结合其对应的有利序列并形成复合物,随后通过一次CZE分离过程,即可同时收集到这两种靶标的复合物。(5)单步毛细管电泳(ssCE)-SELEX^[61]^:在基于在线反应的单步CE-SELEX模式中,混合、反应、分离、检测以及复合物的收集均通过一个单步的在线过程完成。(6)平衡混合物的平衡毛细管电泳(ECEEM)-SELEX^[62]^:将靶标加入到CE的运行缓冲液中,随后将核酸库进样至毛细管柱内;在电泳过程中,与靶标结合的核酸序列与游离的核酸序列因迁移速度的差异而分离,从而实现复合物的分离与收集。(7)毛细管瞬时等速电泳(ctITP)-SELEX^[63]^:通过使用先导电解质和尾随电解质,游离核酸与复合物能够根据二者电泳淌度的差异实现有效分离。

2005年以来,CE-SELEX技术已被应用于各种小分子靶标适配体的筛选(表2)^[55,64⇓⇓⇓⇓⇓-70]^。然而,由于小分子靶标-核酸库复合物与未结合核酸库之间的荷质比差异较小,它们在电泳过程中的迁移速度相近,这使得复合物与未结合的核酸库难以有效分离,进而无法对复合物进行精确的定性及定量分析。因此,通过电泳图谱无法直观地监测到复合物的形成及状态,即无法实现复合物的可视化,这也是传统CZE分离模式难以直接分离和精准收集复合物的主要原因。上述小分子靶标-核酸库复合物的收集过程较为盲目,未充分考虑到复合物与未结合的核酸库无法分离的情况。因此,小分子靶标-核酸库复合物的可视化是适配体成功筛选的关键。

**表2:** CE-SELEX技术在小分子靶标适配体筛选中的应用

Target	Screening steps	Aptamer sequence (5'-3')	Screening round	Affinity determination method	*K*_D_/ (nmol/L)
*N*-Methyl mesopor- phyrin IX (NMM)^[55]^	positive selection	AGCAGCACAGAGGTCAGATGTGCTCGA- CTATTGAGTCAGGGGGTTGGGGCAACA- ATAAGCCCTATGCGTGCTACCGTGAA	3	normalized fluorescence intensity determination	1200.0±100.0^*^
Virginiamycin-M_1_ (VGM-M_1_)^[64]^	positive selection	GCAGAGGGACAGGGAAGATGTAGACAT- CTGTGCTCTTCGC	4	CE-ultraviolet (UV)	49.0
Histone H4-K16Ac^[65]^	counter and positive selection	AGACGTAAGTTAATTGGACTTGGTCGTG- TGCGGCACAGCGATTGAAAT	4	surface plasmon resonance (SPR)	47.0±24.0^*^
Swine anaphylatoxin (C5a)^[66]^	positive selection	TCGCTGTAGCTACAGGGTTTACCCGGTT- GGATGGAT	6	SPR	4000.0
Glycosylated VEGF peptide fragment^[67]^	positive and counter selection	GCACCCTAATGTGCAAGCTAATGCGGAA- TGGGGTCGGTTT	8	surface plasmon reso- nance imaging (SPRI)	2500.0±400.0^*^
Diethyl thiophosphate (DETP)^[68]^	positive selection	GACGGGAGCTTGACACAGTCATTCCTTT- CTAGATGGTGGTT	-	gold nanoparticle colori- metric assay	103.0±14.0^*^
Clenbuterol hydro- chloride (Clen)^[69]^	positive selection	AGGGCCTGGGCTGTTGAGGCAACGTCG- GTTTGTTTATTAA	3	capillary electrophoresis- laser induced fluorescence (LIF)	931.5
Neuropeptide Y (NPY)^[70]^	positive selection	AGCAGCACAGAGGTCAGATGCAAACCA- CAGCCTGATGGTTAGCGTATGTCATTTA- CGGACCTATGCGTGCTACCGTGAA	4	affinity capillary electro- phoresis (ACE)	800.0

VEGF: vascular endothelial growth factor; *: *n*=3; -: not given.

为了解决小分子靶标-核酸库复合物与未结合核酸库难以分离的问题,本课题组^[71]^创新性地开发了动态竞争吸附毛细管电泳(dynamic competitive adsorption capillary electrophoresis, DcaCE)-SELEX模式。DcaCE-SELEX是一种全新的筛选策略,通过增大未结合核酸库与复合物之间的荷质比差异,可以实现二者的有效分离。在我们长期致力于蛋白质与核酸库结合的研究过程中,发现了一系列被称为“核酸捕手”的蛋白质^[71]^,这些蛋白质具有极强的核酸结合能力,并能够无差别地与各种核酸序列结合。基于此发现,我们在筛选体系中引入了“核酸捕手”蛋白质(如转铁蛋白),并将其作为竞争靶标。该“核酸捕手”蛋白质能够有效吸附未与小分子结合或结合程度较弱的游离核酸库,所形成的竞争靶标-核酸库复合物的荷质比远高于小分子靶标-核酸库复合物的荷质比。通过贝克曼荧光电泳图谱可直观监测到竞争靶标-核酸库与小分子靶标-核酸库两种复合物的形成及状态,从而实现小分子靶标-核酸库复合物与未结合核酸库的可视化分离。此外,根据小分子靶标-核酸库复合物的迁移时间,设置精确的收集区段,可以实现小分子靶标-核酸库复合物的精准收集。利用DcaCE-SELEX筛选策略,研究已成功实现7种小分子的适配体筛选。

### 2.2 CE-SELEX技术的优势与应用

目前,CE-SELEX技术已被应用于多类别、性质各异的小分子靶标适配体的筛选,包括抗生素、多肽、毒素、激素、神经递质、代谢产物、兴奋剂和有机化合物等。该技术能够依据小分子靶标的化学特性和生物活性来选择合适的分离模式,通过优化小分子靶标与核酸库之间的结合条件及小分子靶标-核酸库复合物与未结合核酸库之间的分离条件来设计筛选策略。因此,CE-SELEX技术在针对不同结构特征小分子靶标的适配体筛选方面具有广泛的适用性。

CE-SELEX技术能够在自由溶液中筛选适配体,无需对核酸库或靶标进行固定。该技术可以直接对混合物进行分离,使适配体能够从各个位点与靶标结合,避免了因固相固定靶标而导致的筛选偏差。在常用的固定化方法中,小分子靶标或核酸库需通过共价或非共价的方式连接到固相载体上。然而,这种固定过程可能会遮蔽靶标的部分结构,从而导致筛选偏差。例如,Proske等^[72]^将神经肽Y(neuropeptide Y, NPY)固定在磁珠上,并成功获得了NPY的适配体(DP3)。然而,当NPY被分割成不同片段并分别与DP3结合时,DP3仅能够与NPY的部分区域发生相互作用。相比之下,采用CE-SELEX技术对NPY的适配体进行筛选时,可以避免因靶标固定而导致的NPY结构掩蔽问题,从而降低筛选偏差^[70]^。

当采用CZE和DcaCE的分离模式时,CE-SELEX技术可在电泳分离过程中实现复合物的可视化监测。与传统的SELEX技术相比,CE-SELEX技术在电泳分离的同时还能够对复合物的组分进行检测。特别是当采用荧光标记的核酸库时,该技术能够高灵敏地可视化监测筛选过程中结合序列的富集程度及复合物的形成状态,并且支持对各轮次级库与靶标亲和力变化的定量评估。这一特性使得筛选过程变得更加可控,为实时调整筛选条件和循环次数提供了依据,进而提高了筛选效率和准确性。

CE-SELEX技术无需进行负筛步骤,因此筛选效率较高。大多数采用固相基质的筛选方法都需要进行负筛步骤,该步骤涉及将正筛所用的核酸库与用于固定靶标的固相基质进行孵育,旨在去除那些可能与固相基质非特异性结合的假阳性序列^[73]^。由于CE-SELEX技术在自由溶液中进行,无需对小分子靶标或核酸库进行固定化处理,因此省去了负筛步骤,这也是CE-SELEX技术的优势之一。与传统的筛选方法(需8~12轮筛选)相比,CE-SELEX技术仅需1~4轮筛选即可获得高亲和力的适配体,并且每轮筛选中的复合物分离过程仅需约10 min。

CE-SELEX技术所需的进样量小。即便进样量处于nL级水平,CE-SELEX仍能够保持超高的分离效率,显著降低对核酸库和靶标的消耗,因此该技术非常适合用于珍贵靶标的筛选。

### 2.3 CE-SELEX技术存在的问题与挑战

当前在小分子适配体筛选领域,应用CE-SELEX技术的研究相对较少,主要原因有以下几点:(1)CE-SELEX技术的大多数分离模式无法实现小分子靶标-核酸库复合物的可视化监测,导致复合物难以被精确收集,这成为限制CE-SELEX技术在该领域应用的一大瓶颈。(2)小分子靶标-核酸库复合物与未结合核酸之间的电泳淌度差异还会受到电泳溶液性质的影响^[45]^,如CE-SELEX技术对离子强度和pH的要求较为严格,这些特定的条件可能会与SELEX其他步骤所使用的溶液条件发生冲突。(3)由于CE的进样体积较小,单次进样的混合物样品量有限,这导致可有效利用的核酸库序列数变少。因此,通过CE-SELEX技术分离得到的复合物不够完整,存在核酸序列缺失的可能。此外,这一状况可能导致每次筛选的重复性存在差异,进而影响对筛选效果的准确评估。(4)在复合物的分离过程中,溶液的盐浓度和温度会对分离效果产生影响。若复合物与小分子靶标或未结合核酸之间的迁移时间差异过小,可能会导致它们之间难以互相分离。因此,在利用CE-SELEX技术进行筛选的过程中,适配体与小分子靶标之间的最佳结合条件以及复合物与未结合核酸之间的最佳分离条件可能无法统一,导致最终的适配体结合条件难以界定。

## 3 GNP-SELEX技术

### 3.1 GNP-SELEX技术的介绍

GNP-SELEX技术利用金纳米颗粒表面带有的正电荷来吸附ssDNA,从而实现小分子靶标-ssDNA复合物与未结合ssDNA之间的分离。这一特性虽与GO-SELEX技术相似,但二者的机制不同^[74]^。具体来说,金纳米颗粒通过范德华力和强静电相互作用与ssDNA的碱基紧密结合^[75]^,从而吸附ssDNA。然而,金纳米颗粒不易吸附那些已与靶标结合的适配体,因此金纳米颗粒可用于去除未与靶标结合的游离ssDNA。GNP-SELEX技术完全依赖于适配体与靶标之间的亲和力,只有与金纳米颗粒完全分离的适配体才能进入下一轮的SELEX筛选,这一机制显著提高了适配体筛选的灵敏度。GNP-SELEX有望成为未来SELEX技术的一个重要发展方向,并可能成为传统SELEX的优秀备选策略。

最初,GNP-SELEX技术的筛选模式是先将核酸库与靶标孵育,再加入金纳米颗粒共同孵育。在这个过程中,金纳米颗粒会吸附所有未与靶标结合的核酸,而与靶标紧密结合的适配体则不会被金纳米颗粒吸附,随后通过离心操作即可将这两部分有效分离。然而,适配体与靶标之间的相互作用较弱,主要包括静电相互作用、氢键和*π-π*堆叠^[76,77]^;其中氢键的能量约为25~40 kJ/mol,而*π-π*堆叠的能量甚至更低(约为10 kJ/mol),这些作用力远弱于金纳米颗粒对ssDNA的吸附作用力。因此,这种筛选模式主要存在以下两个问题,首先,金纳米颗粒对ssDNA具有较高的亲和力,一些已与靶标结合的序列仍可能被吸附在金纳米颗粒上,从而导致在离心过程中丢失;其次,在多轮筛选过程中,即便添加了靶标,富集后的次级核酸库仍可能继续吸附在金纳米颗粒表面,难以判断筛选是否到达终点。Chatterjee等^[78]^提出在GNP-SELEX筛选过程中采取以下步骤:首先,将核酸库与金纳米颗粒进行孵育,使ssDNA吸附在金纳米颗粒表面;随后加入小分子靶标,此时仅有那些既能与靶标结合且结合亲和力大于其与金纳米颗粒结合亲和力的适配体才能从金纳米颗粒表面释放出来。由于适配体的释放,金纳米颗粒在盐溶液中发生聚集,导致溶液颜色由红色转变为紫色。同时,该过程中金纳米颗粒的分散状态(呈现红色)或聚集状态(呈现紫色)可以作为实时监测候选适配体富集程度的指标,并帮助判断筛选过程的终止。上述筛选模式仅适用于那些在与靶标结合时倾向于完全从金纳米颗粒表面脱离的适配体。目前,GNP-SELEX技术已成功应用于多种小分子靶标的适配体筛选,如表3所示,经过8~11轮筛选后,所获得的*K*_D_为0.017~21.6 μmol/L。

**表3:** GNP-SELEX技术在小分子靶标适配体筛选中的应用

Target	Nucleic acid library type	Aptamer sequences (5'-3')	Screening round	Affinity determination method	*K*_D_/ (μmol/L)
Dichlorvos (DV)^[78]^	DNA	GGAAGAACATGTGAGCAAAAGGCCAGCAAAAGG- CCAGGAACCGTAAAAAGGCCGCGTTGCTGGC	8	ITC	0.850
Amyloid *β*-peptide (A*β*)^[79]^	RNA	UAGCGUAUGCCACUCUCCUGGGACCCCCCGCCG- GAUGGCCA, UUUGGGGUGUCGGGCGAUUUUUA- GGGUUGGGCCAGGCCGU	9	Fluorescence anisotropy measurements	21.6, 10.9
Zinc(II)-protoporphy- rin IX (ZnPPIX)^[80]^	DNA	GGCGGGGGGTTGCTCTACTTGATGATCTGCGCTA- TCGCCGT	11	fluorescence assay	9.53±1.86^*^
Brassinolide (BL)^[81]^	DNA	GCGGATCCCGCGCCGTGCAGAGGGAGACCGC	9	gold nanoparticle colorimetric assay	0.0173
Bisphenol A (BPA)^[81]^	DNA	GGACGCGCGAAGAATATAAGGTGGCCTGGCCGC- GCG	11	gold nanoparticle colorimetric assay	0.0379

*: *n*=3.

### 3.2 GNP-SELEX技术的优势与应用

利用GNP-SELEX技术筛选出的适配体具有较强的特异性和较高的稳定性。Chatterjee等^[78]^通过8轮筛选获得了能够靶向敌敌畏(DV)的适配体(DV2), *K*_D_为0.85 μmol/L。进一步评估了DV2的交叉反应性及其对与DV结构相似农药的区分能力,结果发现,DV2对DV展现出了极高的特异性,回收率约为83%。此外,该研究利用筛选出的DV2,开发了一种基于适配体-纳米酶的比色测定法,该方法对DV的LOD为15 μmol/L,并且该方法在河水和商业苹果汁等复杂环境中依然能保持稳定的检测性能。上述结果表明,GNP-SELEX可作为筛选高特异性小分子适配体及快速构建相应传感器的技术平台。

通过GNP-SELEX技术筛选得到的适配体可用于生物传感研究中的比色检测或化学发光检测。2019年,Li等^[80]^以金纳米颗粒作为分离基质,经过11轮筛选获得了锌(Ⅱ)-原卟啉Ⅸ (zinc(Ⅱ)-protoporphyrin Ⅸ, ZnPPⅨ)的适配体,进一步截短后得到适配体ZnP1.2 (*K*_D_为(9.53±1.86) μmol/L)。研究发现,ZnP1.2对与ZnPPⅨ化学结构相似的*N*-甲基中卟啉Ⅸ(*N*-methyl mesoporphyrin Ⅸ, NMM)表现出了良好的荧光增强效应。此外,在与血红素(hemin)结合后,ZnP1.2的过氧化物酶活性增强。以上结果表明,作为一种功能性适配体,ZnP1.2具有成为发光荧光探针和脱氧核糖核酸酶的潜力,并可作为过氧化物酶的替代品,广泛应用于比色检测或化学发光检测。

GNP-SELEX技术能够在连续的筛选(正向或负向筛选)轮次中直接监测适配体库的富集情况。此外,该技术操作简单、便捷且成本较低,其分离过程仅需离心机,而无需依赖其他大型仪器设备,这对小分子适配体的筛选工作极为有利。Lee等^[81]^选取油菜素内酯(BL)和双酚A(BPA)作为小分子靶标,基于GNP-SELEX技术搭建了一个可视化实时监测平台,实现了适配体的快速筛选。通过监测筛选轮次间的颜色变化(以消光比(*E*_520_/*E*_700_)表示金纳米颗粒的颜色变化),该研究可以很容易地监测每轮筛选中适配体库对靶标(或负筛靶标)亲和力的增减。该方法无需对小分子靶标进行修饰,也无需其他监测步骤,能够快速确定特异性适配体库的富集情况,从而迅速筛选出相应的适配体。

### 3.3 GNP-SELEX技术存在的问题与挑战

GNP-SELEX技术在富集某些小分子靶标的适配体方面存在局限性。Ding等^[82]^研究发现,在采用GNP-SELEX技术筛选腺苷适配体时,从金纳米颗粒上释放的序列数量极少,大多数ssDNA仍被吸附在金纳米颗粒上。由此推断,GNP对ssDNA的强吸附作用可能会阻碍某些小分子靶标与ssDNA的结合,即便发生结合,适配体也可能仍然附着在金纳米颗粒的表面^[34,83,84]^。因此,GNP-SELEX技术可能无法对那些与小分子靶标结合能力较弱的适配体进行筛选,这极大地限制了该技术的推广与应用。

GO-SELEX、CE-SELEX和GNP-SELEX技术的分离介质、筛选机制及优缺点总结于表4。综上所述,针对各种小分子靶标,首先需要评估这些小分子与核酸库的亲和力,然后再选择合适的适配体筛选技术。若小分子靶标与核酸库之间的亲和力相较于GO与核酸库之间的亲和力存在较大差异,则选择GO-SELEX;若小分子靶标与核酸库的结合能力强,则选择GNP-SELEX技术;若小分子靶标能够在自由溶液中与核酸库形成稳定的复合物,且该复合物的荷质比及迁移率与未结合核酸库存在明显差异,则选择CE-SELEX技术。

**表4:** GO-SELEX、CE-SELEX和GNP-SELEX技术的分离介质、筛选机制及优缺点

Screening technique	Separation medium	Screening mechanisms	Advantages	Disadvantages
GO-SELEX	GO	*π*-*π* stacking	simple, quick, high throughput, high affinity	spontaneous desorption, mistaken desorption, incomplete separation, cytotoxicity
CE-SELEX	separation buffer	charge-to-mass ratio and migration rate	multiple screening modes, no fixing, high screening efficiency	difficulty in accurately collecting, susceptible to the properties of the solution, difficulty in defining aptamer-binding conditions
GNP-SELEX	gold nanoparticle	van der Waals forces and electrostatic interaction	high specificity and stability, easy and low cost, colorimetric detection and chemiluminescence detection, directly monitorable	excessive adsorption

## 4 适配体特异性评价中对照靶标的选择标准

准确评价适配体的特异性是当前小分子适配体研究所面临的挑战之一。在特异性评价过程中,通常会引入其他小分子作为对照靶标,通过比较小分子靶标-适配体和对照靶标-适配体的结合情况来评估适配体的特异性。其中,对照靶标的选择直接影响特异性评价结果的准确性和真实性。适配体特异性评价中对照靶标的选择主要考虑同源性、特殊的化学修饰及结构相似性。

### 4.1 同源性

对于多肽类小分子靶标,一般选取与目标多肽同源性≥50%的多肽作为对照靶标,由此鉴定出的适配体能有效区分不同种类的多肽靶标来源。例如,人C5a和小鼠C5a均与猪过敏毒素(C5a)具有较高的同源性(分别为70%和67%)^[66]^,因此在对猪C5a的适配体进行特异性分析时,选择人C5a和小鼠C5a作为对照靶标。

### 4.2 特殊的化学修饰

对于有特殊修饰的小分子,通常选择去除特殊修饰或化学结构相似的小分子作为对照靶标,以此来检测适配体对特殊修饰小分子靶标的识别能力。例如,利用ACE方法分别评估适配体与糖基化的血管内皮生长因子(VEGF)肽(16肽)、非糖基化的VEGF肽以及同样经过糖基化修饰的非同源16肽之间的结合亲和力^[67]^,以此实现适配体的特异性评估。此外,该方法还可用于适配体对小分子靶标特殊修饰位置的特异性评估。Williams等^[65]^为了检验赖氨酸16乙酰化的组蛋白H4肽(histone H4-K16Ac)与适配体的特异性,同时使用histone H4-K8Ac与适配体进行结合实验。表面等离子体共振(SPR)分析结果表明,与histone H4-K16Ac相比,适配体对histone H4-K8Ac的选择性提高了2400倍,说明该适配体具有位置特异性。

### 4.3 结构相似性

对于有机化合物类的小分子靶标,通常选择与靶标具有相似分子结构和相近分子质量的有机化合物作为对照靶标。杨歌等^[69]^将与盐酸克伦特罗(Clen)具有相似分子结构和相近分子质量的沙丁胺醇(Sal, C_13_H_21_NO_3_, *M*_r_为239.31 g/mol)作为对照靶标,利用纳米金比色法验证了适配体与Clen之间的的结合特异性。

## 5 展望

小分子靶标的非固定化核酸适配体筛选技术(主要包括GO-SELEX、CE-SELEX和GNP-SELEX技术)发展迅猛。研究者们将多种新型材料应用于小分子靶标的适配体筛选,并依据靶标的特性设计出多样化的筛选模式。在此过程中,新的筛选技术不断得到开发、优化和完善,从而在筛选效率、特异性、准确性以及高效性等多个方面实现了显著提升。然而,当前可应用的小分子靶标适配体在数量和效果上均远未达到实际需求。随着纳米材料的不断发展,越来越多的新型纳米材料将被用作分离介质,以用于小分子靶标的核酸适配体筛选。理想的纳米材料对核酸碱基的亲和力应介于GO与金纳米颗粒之间,这样既能防止核酸库因自行解吸附而从纳米材料表面脱落导致阳性序列的丢失,又能最大限度地减少因纳米材料吸附性过强而引发的非特异性干扰。此外,通过在筛选过程中引入高通量测序技术,可以监测每一轮次级核酸库序列的进化特征信息;在此基础上,结合生物信息学进行辅助筛选,不仅能够缩短筛选周期,还能提高筛选的成功率。目前,计算机辅助技术是改造和优化适配体序列及结构的主要方法。建立小分子适配体的计算机辅助筛选平台(包括虚拟筛选、结构预测与优化),并结合动力学模拟研究,将为适配体的结构设计、性能优化以及结合机理的深入探索提供重要指导。
